# In silico study of cytochrome-C binding to a cardiolipin-containing membrane

**DOI:** 10.1007/s00249-025-01783-7

**Published:** 2025-07-25

**Authors:** Alessia Muroni, Fulvio Erba, Leonardo Domenichelli, Luisa Di Paola, Federica Sinibaldi, Giampiero Mei, Almerinda Di Venere, Velia Minicozzi

**Affiliations:** 1 Department of Physics, University of Rome Tor Vergata, Via Della Ricerca Scientifica 1, 00133 Rome, Italy; 2 Section of Roma Tor Vergata, INFN, Via Della Ricerca Scientifica 1, 00133 Rome, Italy; 3 Department of Clinical Science and Translational Medicine, Tor Vergata University of Rome, Via Montpellier 1, 00133 Rome, Italy; 4 Unit of Chemical-Physics Fundamentals in Chemical Engineering, Department of Science and Technology for Sustainable Development and One Health, Università Campus Bio-Medico of Rome, 00128 Rome, Italy; 5 Department of Experimental Medicine, University of Rome Tor Vergata, Via Montpellier 1, 00133 Rome, Italy

**Keywords:** Cytochrome C, Membrane binding, Protein conformational dynamics, Superficial roughness, Fractal dimension

## Abstract

Cytochrome C is a key protein involved in electron transport within the mitochondrial respiratory chain and in apoptosis mechanisms. In this work, we provide a detailed theoretical analysis of the binding mechanism between cytochrome-C and a cardiolipin-containing membrane. Molecular dynamics simulations, along with protein contact network and fractal dimension analyses were employed to investigate the structural changes in cytochrome-C during the binding process. Our results suggest that cytochrome-C follows a two-step binding mechanism, starting with a rapid initial interaction, followed by slower conformational rearrangements. We identified two different cytochrome-C conformations at the membrane: a compact, native-like structure and an extended form. The latter, stabilized by Lys72, exhibits a higher binding affinity (≈ 2 kcal/mol) compared to the former. Protein extension also correlates with increased protein-membrane contact and altered heme ring orientation, suggesting that the partial unfolding of cytochrome-C could be crucial for its peroxidase activity and its role in apoptosis. These findings enhance the understanding of the cytochrome-C’s membrane interactions and its diverse functions.

**Supplementary Information:**

The online version contains supplementary material available at 10.1007/s00249-025-01783-7.

## Introduction

Cytochrome-C (cyt-C) is a little, globular, and soluble hemeprotein, which takes part in mitochondria oxidative phosphorylation of healthy cells. Unlike the other cytochromes present in the four complexes of the respiratory chain, cyt-C is neither located in the transmembrane section nor in the peripheral area of these large protein aggregates. Instead, it moves through the intermembrane space, shuttling electrons from complex III to complex IV, to which it temporarily binds for electron transfer. Despite its simple structure and small size, cyt-C may also perform additional functions by adopting different conformations in response to local conditions dictated by the cell’s physiological state. These'moonlighting'activities have significant implications on cell life. For instance, in the presence of apoptotic signals, the protein is induced to directly bind the mitochondrial external membrane and to assume a cardiolipin peroxidase activity, gaining access to the cytosol. This event triggers a series of other processes, which include caspases initiation (i.e. apoptosis) and/or its translocation in the nucleus, to regulate nucleosome setting up (Alvarez-Paggi et al. 2017). For such reason, the mechanism of interaction between cyt-C and the membrane has aroused a great interest and, since the early nineties, a huge number of measurements have been performed to unravel the steps that characterize this process at the molecular level. The prevalence of basic residues (especially lysine) on its external surface makes cyt-C an essentially positively charged molecule, particularly suitable to interact with the negative moiety of membrane phospholipids. Indeed, three of the four putative binding sites of cyt-C on synthetic vesicles contain at least one lysine residue (Lys 22, Lys 60, Lys 72–73), suggesting that electrostatic interactions play a major role in linking the protein to the double layer of liposomes.

As demonstrated by the studies of Englander and co-workers, cyt-C is structurally organized in five folding units (addressed as “foldons”) which progressively assemble and stabilize each other during a stepwise process (Maity et al. 2004, 2005). With the exception of the first foldon, which is highly structured (containing two α-helices), the other units are quite flexible, being characterized by portions of three long Ω-loops that contain approximately 40% of the cyt-C residues. These loops, addressed as proximal, central, and distal (residues 20–30, 40–57, 71–85, respectively) display enhanced structural dynamics (Krishna et al. 2003). It has been shown that their flexibility accounts for functional conformational changes (Krishna et al., 2003), leading to cyt-C peroxidase activity in apoptosis (Hoang et al., 2003; Deacon et al. 2018). Furthermore, they might play a crucial role in driving and stabilizing the interaction of cyt-C with cardiolipin-containing membranes, as recently suggested by molecular dynamics simulations (Muroni et al. 2024). Such feature is particularly important, as natural point mutations occurring in cyt-C loops produce alterations in the mitochondria electron transfer process (Deacon et al. 2020), the onset of thrombocytopenia (De Rocco et al., 2013; Muneeswaran et al., 2017) and an increased level of apoptotic (Karsisiotis et al. 2015; Fellner et al. 2021) and peroxidase activity (Deacon et al. 2017; Samsri et al., 2021).

In the present study, we have investigated through molecular dynamics (MD), fractals dimension (FD), and protein contact network analysis (PCN) the topological features of cyt-C structure upon binding to a cardiolipin-containing membrane. The results indicate that the protein amino acids may be divided into two clusters, one of which contains the two longest Ω-loops involved in membrane binding. The interaction with the bilayer affects both the cyt-C surface roughness and its tertiary structure, producing two different configurations in which the position of the heme ring displays a different orientation and accessibility from the outside. Such a finding may provide new insights into the moonlighting activities of cyt-C.

## Materials and methods

### MD simulations

We performed classical MD simulations of three replicas (A, B and C in the following), each differing in the initial position of the wild-type Cyt-C (PDB ID: 2N9J) relative to a POPC/POCL (half POPC and half POCL) membrane consisting of 256 molecules (128 in the upper layer and 128 in the lower layer), built and parametrized with the help of the CHARMM-GUI server (Brooks et al. 2009; Lee et al. 2016).

The open-source software GROMACS (Van Der Spoel et al. 2005; Abraham et al. 2015) was used employing the CHARMM36 force field (Huang et al. 2017) for the Cyt-C and the membrane, while the parameters for the heme group were taken from (Autenrieth et al. 2004).

We built tetragonal simulation boxes measuring (11.26 × 11.26 × 20.00) nm^3^, considering periodic boundary conditions and enough space along the z-direction to avoid interactions with the system images. We solvated each replica with TIP3P water molecules and added Na^+^ and Cl^−^ ions to neutralize the systems, obtaining replicas with approximately 2 × 10^6^ atoms each. The steepest descent algorithm was used to minimize the systems’ potential energy. Afterwards, each system was equilibrated for 1 ns at a temperature of 300 K, first in the NVT ensemble and then in the NPT ensemble. The subsequent production runs consisted of 500 ns long NPT simulations at 300 K where the MD equations of motion were integrated with a time step of 2 fs. Nose–Hoover thermostat and Parrinello-Rahman barostat were selected to keep temperature and pressure constant at 300 K and 1.0 bar, respectively. The LINCS algorithm was used to constrain bonds involving hydrogen atoms. The sphere’s radius defining the list for pairwise interactions and the van der Waals interaction cutoff radius were fixed at 1.2 nm. For long-range electrostatic interactions, the Particle Mesh Ewald method was utilized with a real space cutoff of 1.2 nm. Initial velocities were derived from a Maxwell distribution centered around the desired temperature.

### DG calculations

The binding affinity (ΔG) of Cyt-C and POCL membrane was calculated, for each of the three replica, by taking as a reaction coordinate the distance ξ between the center of mass of the Cyt-C molecule and the center of mass of the POCL membrane. Following the consolidated *Umbrella Sampling* procedure (Kumar et al. 1992), the center of mass of the Cyt-C was initially pulled along the ξ coordinate from its starting position, ξ_0_ (Cyt-C bound to the POCL membrane) to a final value, ξ_F_, where the interactions between the cytochrome and POCL can be considered negligible. This pulling procedure generated a trajectory consisting of a series of configurations along the ξ coordinate. From this trajectory, 25 configurations (referred to as windows) were selected to span the [ξ_0_, ξ_F_] interval. In each window, the distance between the centers of mass of Cyt-C and the POCL membrane was restrained at ξ_n_ = ξ_0_ + nΔξ, with n = 0, …, 24, by adding a harmonic potential to the system's Hamiltonian, using a force constant of k = 1000 kJ mol⁻^1^ nm⁻^2^.

For each window, a 50 ns MD simulation of the biased system was performed and subsequently analyzed. In this way, it is possible to obtain the Potential of Mean Force (PMF) profile in a set of 25 “overlapping windows” each centered around one of the ξ_n_ values spanning the [ξ_0_, ξ_F_] interval. The data from all windows were combined using the Weighted Histogram Analysis Method (WHAM) algorithm to compute ΔG. The free-energy computations described above were carried out using the gmx wham routine implementing the WHAM algorithm (Hub et al. 2010).

All numerical simulations were executed using the CINECA Consortium's Leonardo cluster.

### FD calculation

FD has been computed according to:

$$2-FD=\frac{dlog\left(SASA\right)}{dlog\left(PR\right)}$$

(Lewis et al., 1985), where SASA represents the Solvent Accessible Surface Area and PR is the probe’s radius.

A Python script has been developed to calculate FD. An array of probe radii ranging from 1.0 to 2.0 Å (step 0.2 Å) has been used to calculate SASAs with different sensitivity. The data, reported as log(SASA) vs log(PR), have been fitted using linear regression to obtain the FD values for the t = 0 and t = 500 ns MD’s frames (see Figure S1 in Supplementary Material).

### PCN analysis

We applied the PCN method (Di Paola et al. 2021) to carry out network clustering and to compute the betweenness centrality (BC).

The PCN is built up starting from the PDB file, extracting the coordinates of α-carbons. The PCN nodes are single residues and links are defined between pairs of residues if their distance is comprised between 4 and 8 Å (the distance is computed considering the position of α-carbons). The adjacency matrix is the mathematical representation of the network; it is a binary matrix where the value is 1 if there exists a link between the corresponding residues. According to the adjacency matrix, it is possible to define the node degree as the number of links a given node participates in. The minimum number of links connecting to residues is the shortest path in the network, and the BC is defined as the number of shortest paths passing by a given node. High values BC identify nodes that play a role in transmitting signals throughout the protein molecular structure. BC is calculated according to the equation:

$$BC\left(i\right)={\sum }_{v\in V,v\ne i}{\sum }_{u\in V,u\ne i}\frac{{\sigma }_{uv}\left(i\right)}{{\sigma }_{uv}}$$

where ${\sigma }_{uv}$ is the number of shortest paths connecting nodes u and v and ${\sigma }_{uv}\left(i\right)$ shortest paths numbers connecting the two nodes through node *i*.

Network clustering is computed according to the Laplacian matrix, defined as the difference between the degree matrix (a diagonal matrix whose diagonal is the degree vector) and the adjacency matrix (Tasdighian et al. 2014). The spectral decomposition of the Laplacian matrix identifies the Fiedler vector, *i.e.*, the eigenvector corresponding to the second minor eigenvalue of the Laplacian matrix. The components of the Fiedler vector are used to partition network nodes into clusters. The partition into two clusters is applied on the basis of the values of the components of the Fiedler vector, whether lower or larger than the average value of the components. Upon network partition into clusters, it is possible to define for each node the participation coefficient (P) as:

$$P_{i} = 1 - \left( {\frac{{k_{{{\mathrm{si}}}} }}{{k_{i} }}} \right)^{2}$$

Where *k*_*i*_ is the node degree, whereas *k*_*si*_ is the number of links the *i*-th node shares with nodes belonging to its own cluster (intra-cluster degree). High values of P (close to 1) reflect the node’s role in bridging communication between clusters.

## Results

### Cluster analysis of cyt-C

The structural features of simulated cyt-C have been investigated through a PCN analysis (Rosignoli et al. 2023). Briefly, the frames generated in three different molecular dynamics runs (originated from the 2N9J pdb file, Muroni et al. 2024) have been characterized by a clustering procedure that identifies all the nearest neighbors of each amino acid contained in the protein structure, at time t. The analysis, extended to all simulations revealed that two topologically distinct groups are sufficient to satisfactorily describe the protein molecule. As a matter of fact, a three-cluster analysis yielded highly variable results over the time course of the simulation and across the three runs, without revealing any clear or consistent rationale (see Figure S2 in Supplementary material).

**Fig. 1:**
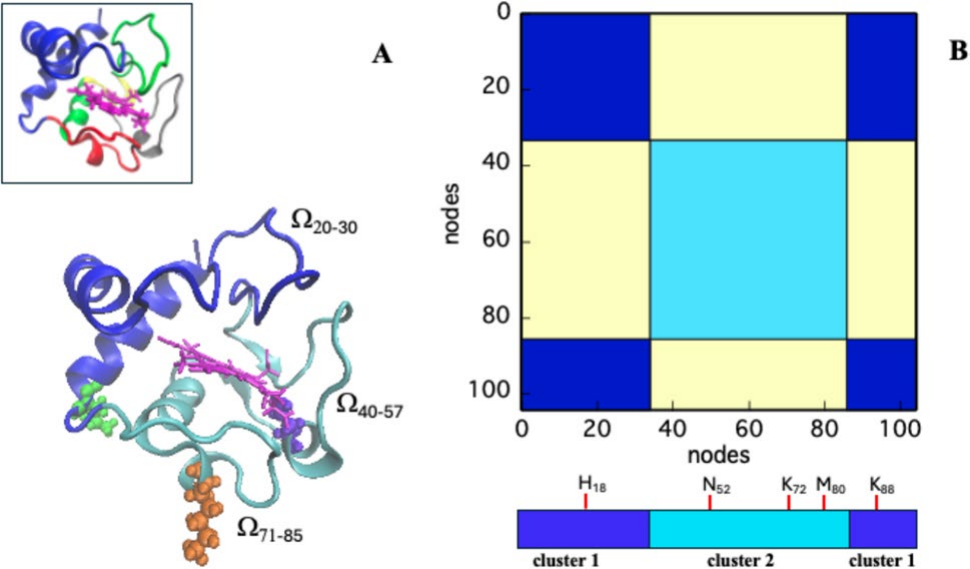
Graphical rendering of cyt-C structure. In panel A, the cartoon of free cytochrome-C is reported in two main colors (blue and cyan) which represent the two clusters obtained in a PCN analysis, at t = 0 ns. The heme structure is reported in magenta. The small inset in the square (upper corner, left) represents the same protein structure colored according to the five folded units reported in the literature (blue, green, yellow, red, and gray, Hu et al. 2016). In panel B, the spectral clustering representation of the two clusters (blue and cyan) identified through the partition routine is shown (Rosignoli et al. 2023). In the bottom, the position within the two clusters of axial Fe-ligands, His18 and Met80, and Asn52, Lys72, and Lys88 are reported

The two amino acid groups belonging to the two clusters are reported in the cartoon of Fig. 1A in blue and cyan, respectively. As shown in the corresponding spectral diagram presented in Fig. 1B, the first cluster (CL1, in blue, residues 1–34 and 86–104) contains one of the two axial ligands of the iron (namely His18), and loop Ω_20–30_, thus accounting for the initial and final segments of the cyt-C primary structure. The comparison with the inset reported in Fig. 1A demonstrates that this cluster corresponds roughly to the first two foldons characterizing the energy landscape and folding pattern of cyt-C (Hu et al. 2016), that is the initial and final α-helices. Thus, with the exception of Ω_20–30_, CL1 is characterized by a high content of secondary structure. The second group of residues contains, instead, the sixth iron-coordinated ligand (Met 80) and the longer loops Ω_40–57_ and Ω_71–85_ (cyan, Fig. 1A, B), which are very mobile segments. Such partition in two clusters is quite sharp, as no “fringes” of opposite color intersect the uniform squares reported in Fig. 1B (Di Paola et al., 2012; Di Paola et al., 2015). This feature would suggest that, from the topological point of view, the two clusters appear and probably act as two individual units. The cartoon shown on the left side of Fig. 1 visualizes more accurately this idea, the heme group appearing as the structural delimiter of the boundary surface between the two modules.

### Details on cyt-C binding with a model membrane: kinetics and binding energy

**Fig. 2:**
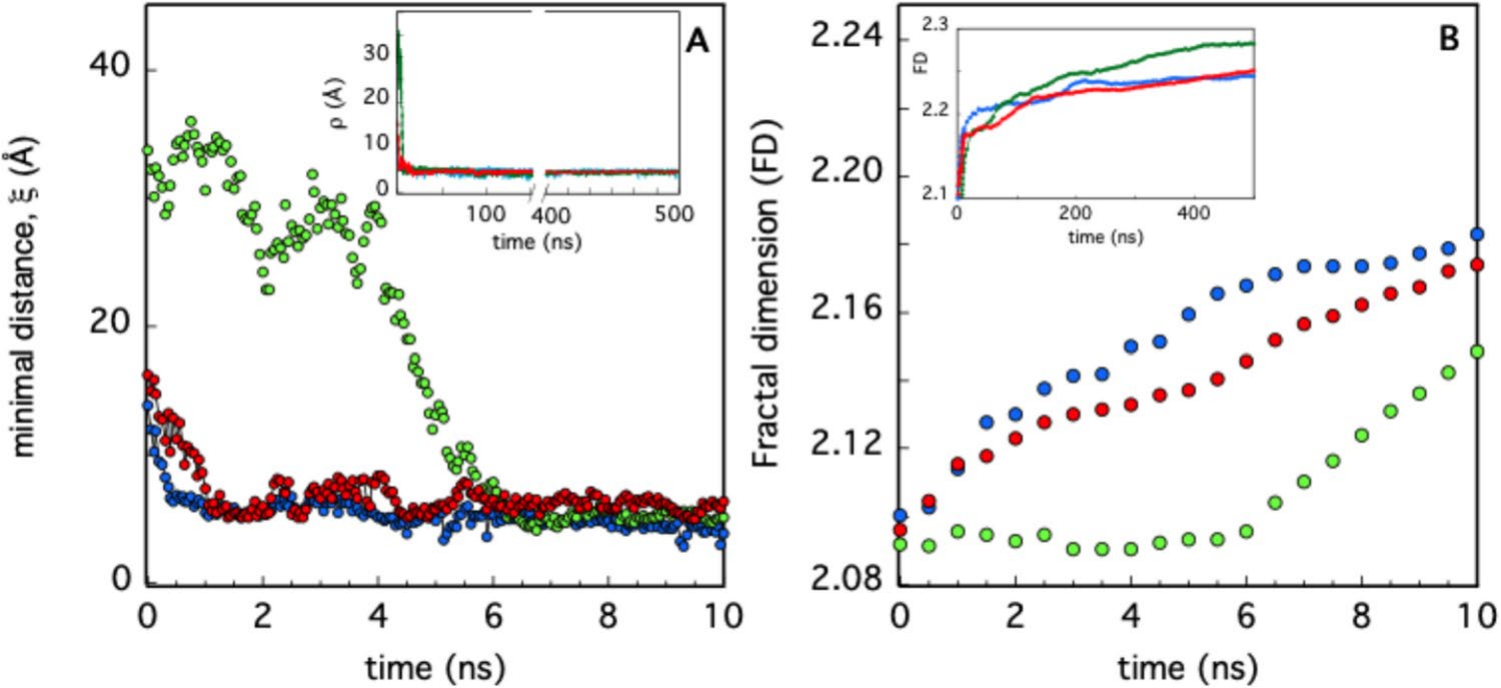
Kinetics of cyt-C membrane binding. In panel A, the minimal distance from the membrane surface is reported in the interval 0–10 ns of simulation, for three independent runs (**A**, in blue; **B**, in green; **C**, in red). In the inset the whole simulation is shown, to provide evidence that a stable interaction takes place. In panel B, the calculated FD is shown at the same time interval (in the inset) for the whole simulation time. The colors correspond to those used on the left side of the figure

The approaching of cyt-C to a model membrane has been monitored by plotting the minimal distance, ξ, between the protein and the bilayer as a function of time (Fig. 2A). The three independent simulations, obtained starting from different initial distances and cyt-C orientations, demonstrate that the interaction takes place in a few nanoseconds and that the protein remains closely in contact with the membrane for the rest of the time (Fig. 2A, inset). Although ξ is a useful parameter to describe the time-dependent features of the protein-membrane interaction process, it does not provide any details on the local conformational changes induced in the protein’s outer shell during the interaction process. For instance, the overall roughness of the cyt-C surface is expected to be strongly influenced by the electrostatic and hydrophobic forces established with the dual-natured membrane components (namely phospholipid heads and tails). Several studies have demonstrated that the surface roughness of large biological macromolecules may be efficiently described by fractals and that, in particular, the FD of globular proteins is typically in the range of 1.7–2.3 (Daniel et al. 1999; Tordoff et al., 2014). We have therefore evaluated, for each step of the MD simulations, the FD of cyt-C, isolating the protein from the membrane, and the result is plotted in Fig. 2B. In the first phase (t ≤ 10 ns), the FD value rapidly increases, as the protein approaches the membrane. This is particularly evident in the second simulation (reported in green in Fig. 2), in which the large, initial protein-membrane distance (more than 30 Å) entails a lag-phase of about 6 ns, which is absent in the faster binding process observed in the other two cases (Fig. 2A and B). A second, slower increase in FD is subsequently observed before an asymptotic value is reached in each simulation (Fig. 2B, inset). Such behavior would suggest that local adjustments of the cyt-C external tertiary structure occur after binding, due to the optimization of the protein-membrane interacting interface.

**Fig. 3:**
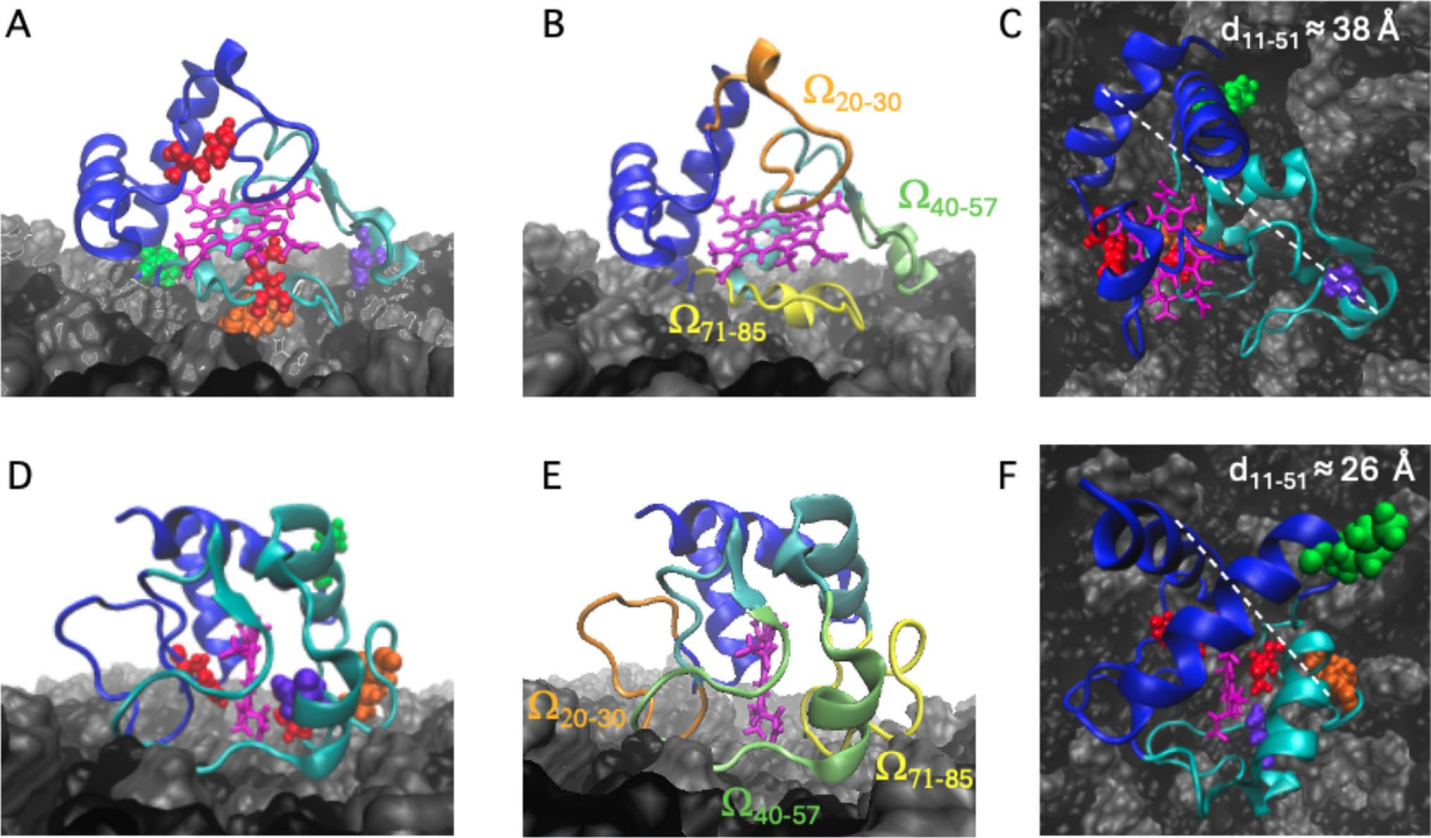
Cyt-C membrane binding configurations at t = 500 ns in simulations A (panels **A**, **B**, **C**) and C (panels **D**, **E**, **F**). In all panels the two clusters are reported in blue and cyan; the heme ring is in magenta; the membrane components, POPC and CL, are shown in gray and black, respectively. In panels A and D, the side chains of Asn52 (green), Lys72 (orange), and Lys88 (purple) are represented as van der Waals (VDW) spheres. The two axial Fe ligands (His18 and Met80) are also shown in red. In panels B and E, the same configuration as in panels A and D is presented, highlighting the three Ω-loop positions (in yellow, lime, and orange). In panels C and F, the corresponding top view images are reported, with the distance between the α-carbons of Ile11 and Ala51 shown in white

The final configurations obtained at t = 500 ns have been reported in Fig. 3, for both simulation A and C (simulation B yielded the same result as simulation A, data not shown). The cartoons indicate that two quite different binding arrangements yield. In the first one (run A, Fig. 3, upper panels), three amino acids appear in close contact with the bilayer, namely Asn52, Lys72, and Lys88. The first two residues belong to the second cluster (a.a. 35–85), while Lys88 resides in the first one. The main characteristics of such a configuration are the heme ring orientation, which is almost parallel to the double layer, and an elongated shape compared to the initial conformation (Fig. 3A). Both features are not observed in the second run (simulation C), in which the compact, globular shape of the crystallographic file is retained (Fig. 3C). The interaction with the bilayer mainly involves the most mobile cyt-C sections (Fig. 3B, E), although relevant differences occur between simulations A and C. In the first case, the major contribution arises from the whole Ω_71–85_ segment, which lies at the protein-membrane interface, while minor support comes from the far end of the Ω_40–57_ loop (Fig. 3B). Again, it is worth noting the role of Lys72 (and partly) of Asn52 in anchoring the cyt-C molecule to the lipid bilayer. In the case of simulation C, instead, all three Ω-loops display some contact points with the membrane, but none of them is fully involved in the binding interface (Fig. 3E).

The different shapes assumed by cyt-C in the two simulations are particularly evident comparing the top views reported in Fig. 3C and F, in which also a quantitative measurement of the larger conformational change occurring in simulation A has been reported. For such purpose, we selected two residues located on protein opposite sites, namely Ile 11 and Ala 51, and measured the distance between their α-carbons, d_11–51_, for each simulation. As indicated in Fig. 3C and F the structure obtained in simulation A displays an increment of ≈ 12 Å with respect to the compact configuration occurring in simulation C, thus suggesting a more open conformation, flattened on the membrane surface.

**Fig. 4:**
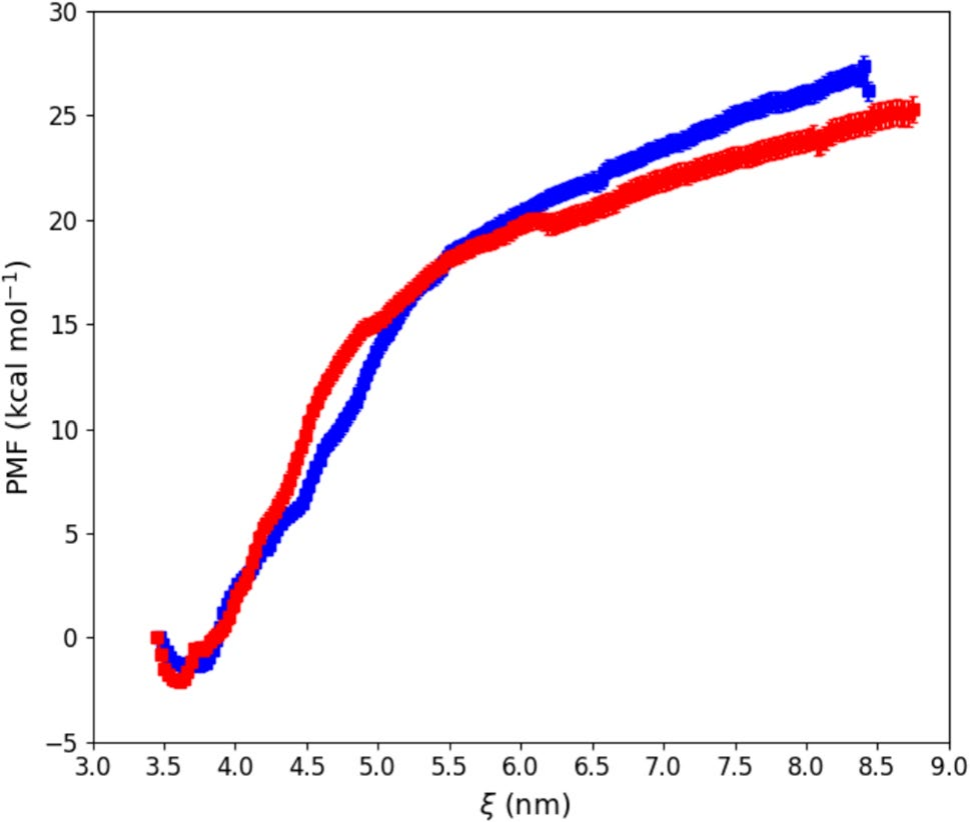
PMF profile for simulation A, blue, and C, red

Figure 4 shows the PMF profiles obtained from simulations A and C, from which the difference in binding free energy between the two systems can be estimated. The resulting value of ΔΔG is approximately 2 kcal/mol, indicating that Cyt-C is slightly more strongly bound to the POCL membrane in simulation A than in simulation C.

### Details on cyt-C binding with a model membrane: effects on the protein tertiary structure

**Fig. 5:**
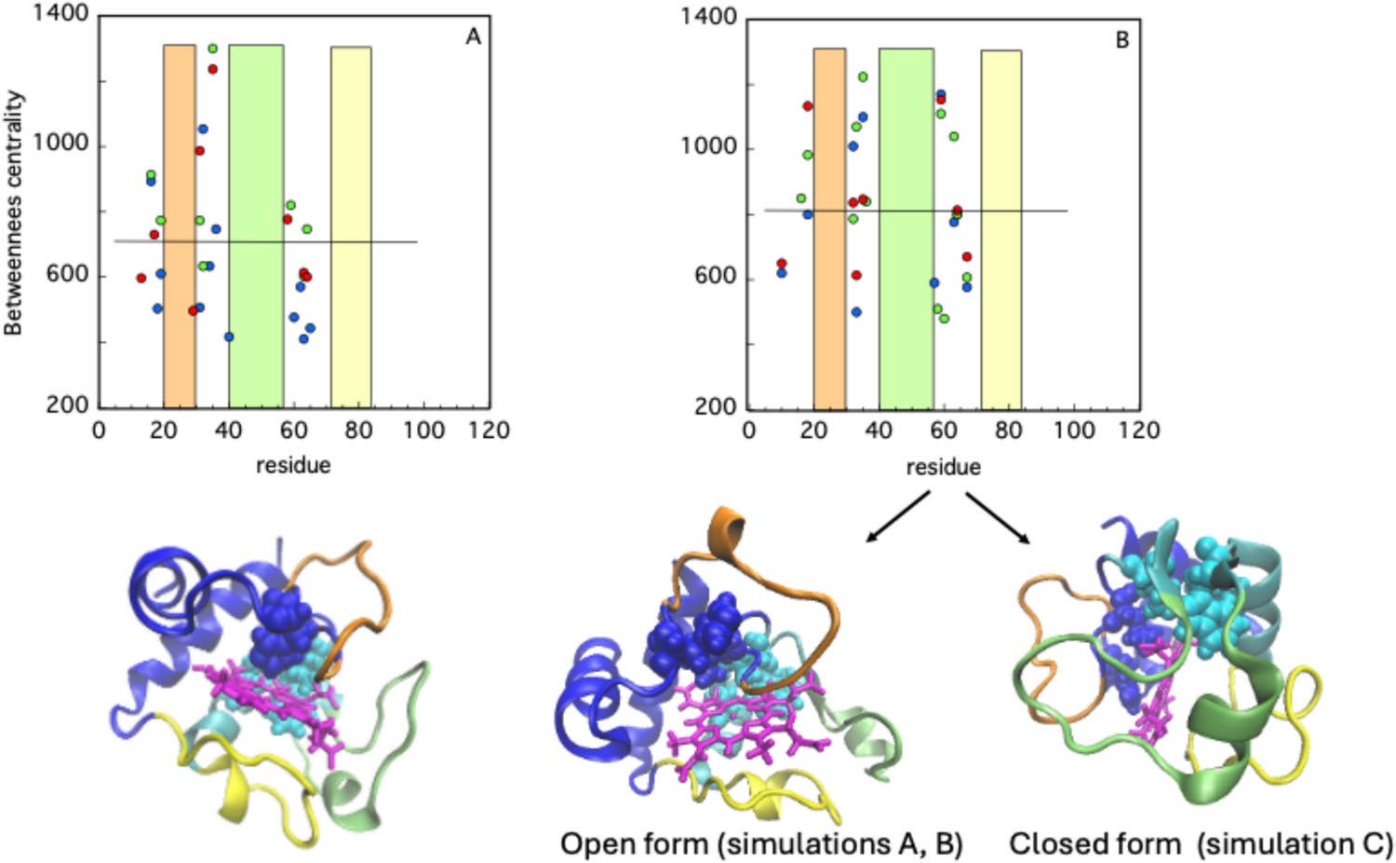
Cyt-C BC at t = 0 (panel **A**) and t = 500 ns (panel **B**) for the three simulations (blue, green, and red circles). The average values are indicated by horizontal lines. In the corresponding cartoons (oriented as in Figs. 1 and 3) the residues with the highest BC (i.e. His 18, His 32, Leu 35, Trp 59, Leu 64) are reported as VDW spheres in blue (for CL1) and cyan (for CL2). The rectangles (in panels **A** and **B**) and the segments in orange, green, and yellow (in the three cartoons) correspond to the protein Ω-loops, as depicted in Fig. 3B and E

The FD trend observed during cyt-C interaction with the lipid bilayer (Fig. 2B) suggests that changes in the protein surface occur. However, it does not indicate whether, or to what extent, the protein interior is affected by its association with the membrane. To quantify a possible perturbation of the protein inter-module contacts, a PCN analysis has been carried out. In particular, we took into account two specific parameters that efficiently describe a protein structure, namely the betweenness centrality and the participation coefficient (Di Paola et al., 2015; Patel et al. 2024), as described in Materials and Methods. The high BC values obtained for each simulation are reported in Fig. 5A (at t = 0 ns) and 5B (at t = 500 ns). In both cases, none of the residues belonging to the three Ω-loops display a relevant BC, as well as the amino acids constituting the two peripheral α-helices. The residues with the largest BC are instead concentrated around one side of the heme ring (Fig. 5A) and lie at the boundary between the two clusters, a position that is conserved when the protein is in contact with the lipid bilayer (Fig. 5B). Interestingly, the average BC value (referred to all residues) is higher in both membrane-bound conformations compared to that of free cyt-C. Such a feature would suggest that their role in bridging the two modules increases under the mechanical stress exerted by membrane binding. As pointed out before, P gives information on the “collaboration level” among the different modules of a protein structure.

**Fig. 6:**
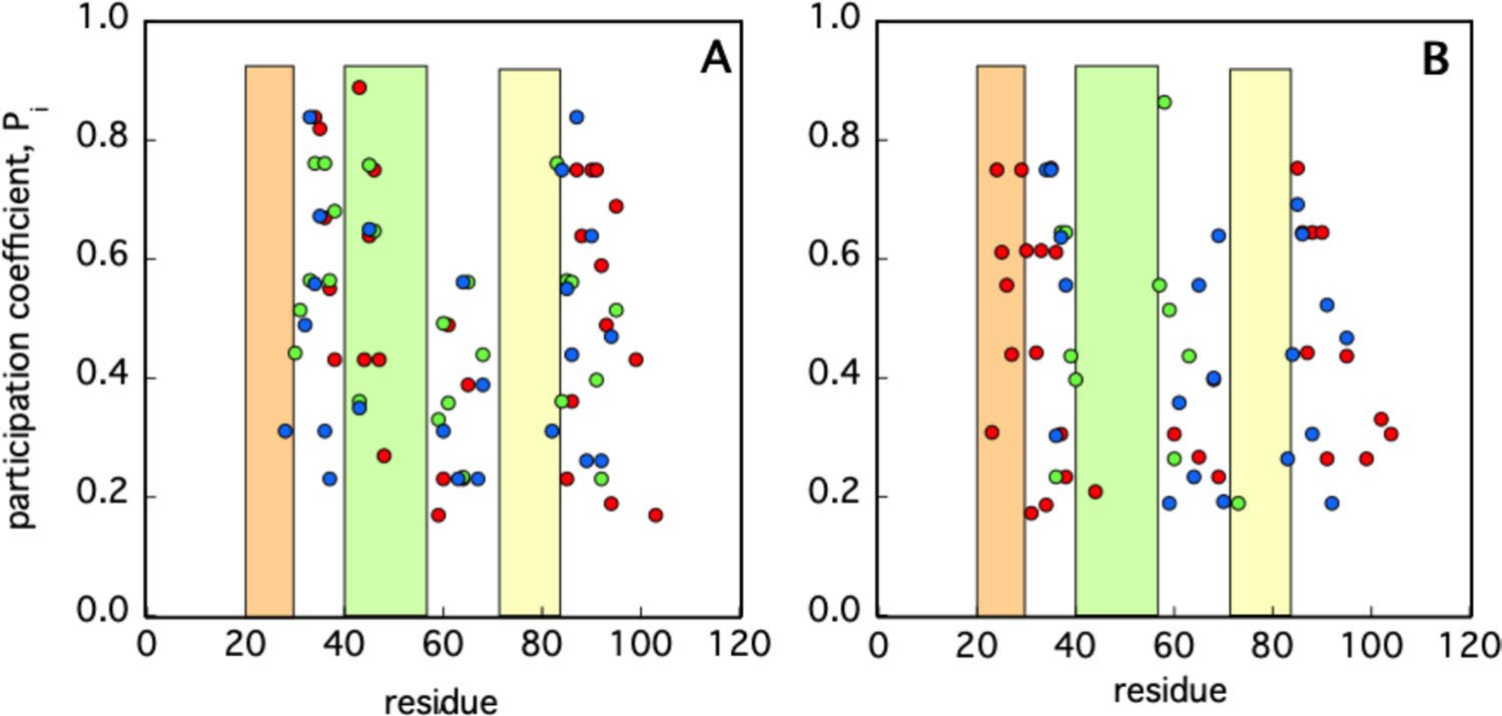
Significant participation coefficient, P, of cyt-C residues for the three simulations (A, blue; B, green; C red circles) at time t = 0 (panel A) and t = 500 ns (panel B). The three colored rectangles circumscribe the Ω loops regions (Ω_20–30_, orange; Ω_40–57_, lime; Ω_71–85_, yellow)

The results for the three simulations of cyt-C are reported in Fig. 6A and B, in the case of free and membrane-bound proteins, respectively. Initially, in all simulations, significant P values are observed only for residues falling within three distinct segments of the protein sequence, namely 30–45, 60–68, and 85–100, (Fig. 6A). Of note, the amino acids contained in loops Ω_20–30_ and.

Ω_71-85_ are characterized by P = 0, while relevant P values (in the range 0.25–0.85) have been obtained only in the case of Ω_40–57_ (Fig. 6A). Thus, within the protein’s most mobile sections, the central Ω-loop is the only one to play a significant role in cluster–cluster communication, when cyt-C is not bound to the membrane. Such a feature no longer holds at t = 500 ns, when almost all residues belonging to Ω_40-57_ display P = 0 (Fig. 6B). The displacement of the points that populated the Ω_40-57_ area at time t = 0 is particularly interesting in the case of simulation C, in which the Ω_20-30_ loop becomes populated and contains about ≈ 30% of the residues with a high participation coefficient (Fig. 6B). Taking into account such result and the position of the Ω_20-30_ loop reported in the cartoon of Fig. 3E, it could be tempting to speculate that this loop might exert a crucial structural role in keeping an intact compact and globular structure, in this particular membrane-bound cyt-C configuration. The role of this loop is confirmed by the effects on the structural dynamics and peroxidase activity of the protein of the mutation T38C (Samsri et al., 2021).

**Fig. 7:**
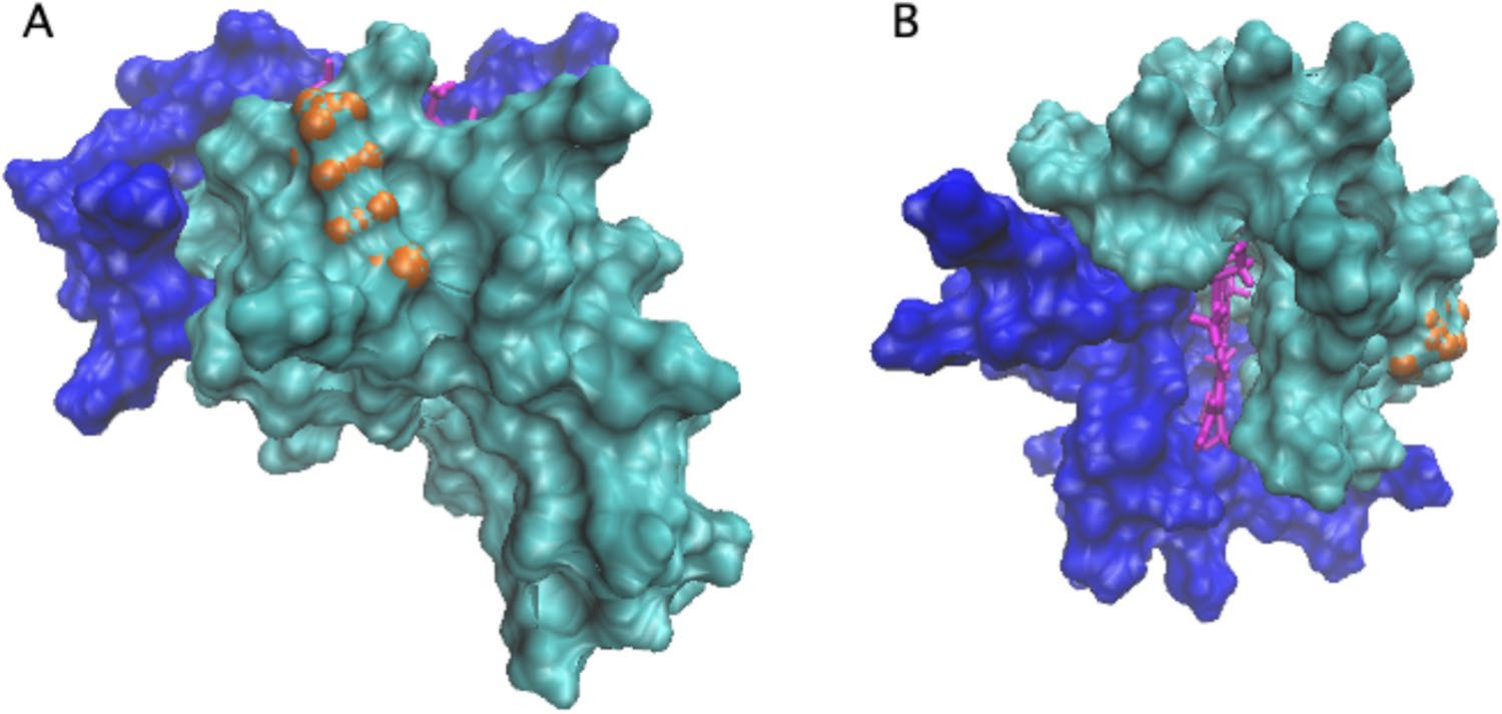
Protein surface in contact with the membrane at t = 500 ns in the case of the open conformation (simulation **A** and **B**, panel **A**) and closed form (simulation **C**, panel **B**). The two clusters, CL1 and CL2, are colored in blue and cyan, respectively, while the heme group is in magenta. The position of Lys72 is represented by orange spheres

Final information on the different configurations assumed by cyt-C upon membrane binding arises from the analysis of the interface between the protein and the bilayer. In the open form (obtained in simulations A and B), most of the protein surface (896 Å^2^) in contact with lipids involves the second cluster, CL2. In this case, the heme group is shielded by the cyt-C matrix (and in particular by the Ω_71–85_ loop), and it is not in direct contact with the membrane surface. On the contrary, for the closed conformation (observed in simulation C) the graphic rendering reported in Fig. 7B indicates that the clusters equally contribute to the shape of the surface interface (710 Å^2^). In this configuration, Lys72 still participates in membrane binding (Fig. 7B), but it makes a minor contribution compared to the open conformation (Fig. 7A). Notably, the accessibility to the heme group is considerably enhanced in the compact form (Fig. 7B), where the Ω_71-85_ loop is rotated by 90° (Fig. 3E) and thus only partially inserted into the membrane. Finally, such an arrangement forces the protoporphyrin into an orthogonal position with respect to the plane of the protein-lipid interface (Fig. 7B).

## Discussion

MD simulations combined with a PCN analysis suggest that the dynamic of cyt-C binding to cardiolipin-containing membranes is complex. The results shown in Fig. 2A, based on the sole minimal distance, ξ, would suggest that the binding process is fast and is completed in a few nanoseconds. Since ξ indicates the average position of the whole protein with respect to the lipid bilayer, it cannot provide further information on possible protein conformational changes occurring in the long-term time range. On the other hand, the occurrence of cyt-C structural conformational changes upon membrane binding has been experimentally documented since 1991 (Muga et al. 1991) and then analyzed in detail by Kinnunen and co-workers (Subramanian et al. 1998). Over the next few years, it was shown that the interaction with the lipid bilayer affects the protein’s tertiary structure, leaving a large part of its α-helices content almost intact (Sanghera et al., 2000). Since the beginning of protein science (in the early 1900 s) it was clear that the tridimensional shape of a protein is strictly correlated to its biological function. The irregular form of the protein surface was immediately recognized as an important discriminant in protein–ligand, protein–protein, and protein-membrane interaction (Ceccarelli et al. 2017, Di Venere et al., 2014). The search for appropriate parameters characterizing such superficial roughness had a breakthrough in 1987 with the introduction of the protein “fractality” (Åqvist et al., 1987), which is the association of a fractal dimension to proteins. The advantage of a fractal analysis is that it provides a quantitative description of the spatial heterogeneity of large macromolecular structures (Torodoff et al., 2014). In the case of proteins, fractals have been already used to predict the location of small functional sites (Pettit et al., 1999), to describe the local tertiary structure at the surface (Daniel et al. 1999) or in their interior (Banerji et al., 2011), to quantify the roughness of receptors surface (Kaczor et al. 2012) and that of enzymatic active sites (Banerji et al., 2013). The characterization of cyt-C binding dynamics in terms of fractal dimension demonstrates that the process is more complicated than what was observed through the minimal distance analysis. The trends for the three simulations reported in Fig. 2B indicate that the roughness of the cyt-C surface increases as the protein gets in contact with the bilayer, following a two-step mechanism, in which a burst phase of a few nanoseconds is followed by a much slower dynamic (Fig. 2 inset). Such behavior suggests that the interaction with the membrane quickly ripples the cyt-C surface, which at a later stage undergoes minor structural re-adjustments, to find a stable conformation. As shown in Fig. 3, this search produces both a partially open and a more compact conformation at t = 500 ns. In the first one, the protein is anchored to the membrane through the distal omega loop (Ω_71–85_) and, in particular, via Lys72 (Fig. 3A and B). The second conformation retains, instead, the globular shape of the crystallographic structure, involving residues of all omega loops in membrane binding (Fig. 3D and E). The co-existence of both native-like and partially unfolded cyt-C conformations on a cardiolipin-containing membrane has been observed with several experimental methodologies, in the past years (Hanske et al. 2012; Muenzner et al., 2014; Pandiscia et al., 2015a; Pandiscia et al., 2015b). A widely accepted hypothesis on the origin of cyt-C unfolding on the membrane surface involves electrostatic interaction between the negatively charged phospholipid heads and (some) protein lysine residues (Schweitzer-Stenner 2018). The extent of such structural unfolding, as well as the region of the protein involved in this conformational transition, are still not completely established. Both evidence of a rather extended unfolding (Hong et. al. 2012; Hanske et al. 2012) and a more modest non-native conformation (Pandisha et al., 2015a; Pandisha et al., 2015b) have been reported in the literature. Such discrepancies might arise from both ambient conditions (e.g., pH and salt concentration) and, more importantly, from cyt-C crowding on the membrane surface (Oellerich et al., 2004). Although the last effect cannot be accountend for in MD simulations, a non negligeable difference in the binding energy (≈ 2 kcal/mol) between the two configurations has been found even in silico (Fig. 4). This result suggests that the cyt-C-membrane affinity is larger in the extended protein conformation. A possible rationale may be inferred by examining the surface that comes into contact with the lipid bilayer in the open cyt-C form. As shown in Figs. 3 and 7 there are at least three factors that might contribute to better stabilizing the extended form: (i) the larger area involved in the interaction (Fig. 7); (ii) the deep insertion of Lys72 (Figs. 3A and 7a); (iii) the participation of a whole Ω-loop (namely Ω_71–85_). This last point is particularly important because loops participate in membrane binding in both protein conformations (Fig. 3B and E). According to the PCN analysis, loops are scarcely relevant for structural cluster–cluster interaction, both in free- and membrane-bound cyt-C (Fig. 5A and B, respectively). Instead, recent experimental results strongly suggest that they could play a major role in the modulation of peripheral interactions between the protein and the membrane (Li et al., 2021), probably because they are the less stable and most flexible structures of the cyt-C modular architecture (Maity et al. 2004, 2005; Hu et al. 2016). Such a hypothesis is also supported by the reduced capacity in membrane binding observed in pathological mutants (G41S and Y48H) both occurring in the Ω_40–57_ cyt-C omega loop (Muroni et al. 2024).

The influence that the membrane exerts on the cyt-C structure has been the focus of several studies, in the past years. While local changes in the environment of Met80 are universally recognized as the source of cyt-C peroxidase activity (Alvarez-Paggi et al. 2017), the origin of the protein ability in pore formation (and thus in apoptosis induction) is still a rather debated question. Both minor structural changes (O’Brien et al., 2015) and protein unfolding (Bergstrom et al. 2013; Kitt et al. 2017) have been invoked as the necessary steps to initiate protein permeabilization. The different orientation of the heme ring found in independent MD runs (Fig. 7) correlates with the extension of the protein-membrane contact surface, thus suggesting that at least partial unfolding might indeed characterize a fraction of cyt-C membrane bound molecules. These findings would support the idea that binding to cardiolipin-containing membranes is not homogeneous (Pandisha et al., 2015a; Pandisha et al., 2015b) and that the presence of multiple protein conformations must be taken into account in both in vitro and in vivo measurements.

## Supplementary Information

Below is the link to the electronic supplementary material.

Supplementary file1 (DOCX 347 KB)

## Data Availability

The data that support the findings of this study are available from the corresponding authors upon reasonable request.

## Abbreviations

cyt-C: Cytochrome-C
MD: Molecular dynamics
CL1: Cluster #1
CL2: Cluster #2
PCN: Protein contact network
FD: Fractal dimension
