# Supplementary material for: In silico study of cytochrome-C binding to a cardiolipin-containing membrane
Source: Eur Biophys J. 2025 Jul 25;55(2):263–73. doi: 10.1007/s00249-025-01783-7 (PMC13109120; doi:10.1007/s00249-025-01783-7)
Supplement: Supplementary file 1 — Supplementary file1 (DOCX 347 KB) [file 249_2025_1783_MOESM1_ESM.docx]

**Supplementary Material**

**Figure S1.** Dependence of the accessible surface area (SASA) on the probe radius (PR) in a double logarithm scale for t = 0 ns (squares) and t = 500 ns (triangles), in the case of simulation B. The corresponding FD values are 2.116 and 2.256 respectively, calculated as: FD = 2 – m, where m is the fit slope, as described in Materials and Methods.


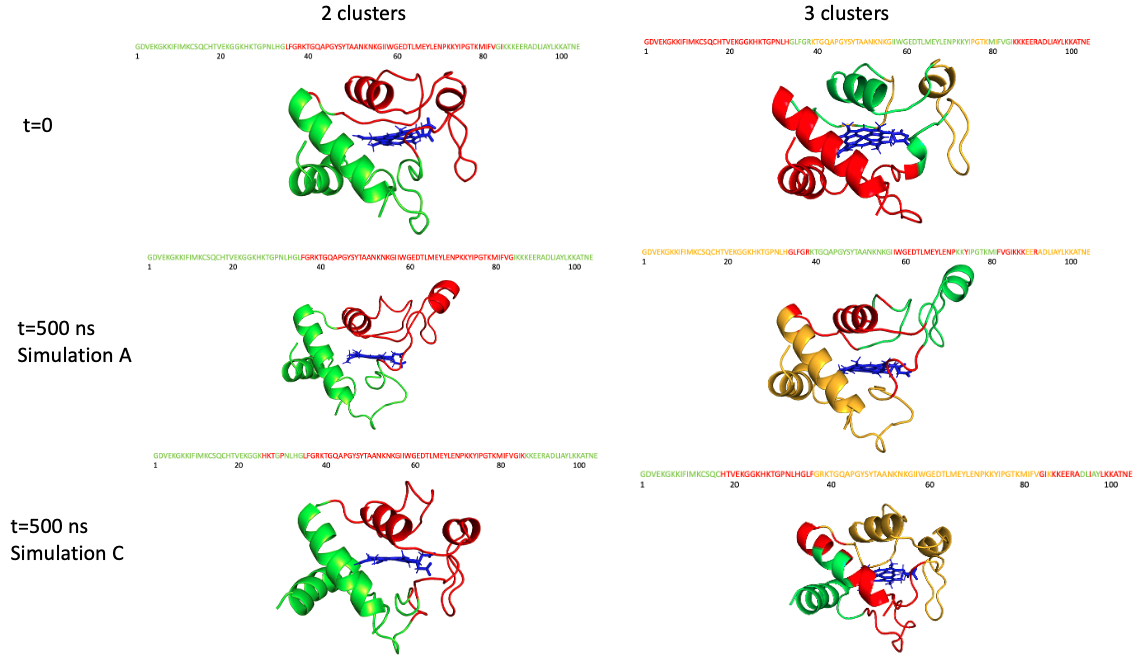


**Figure S2.** Comparison of two (red and green) and three (red, yellow) and green cluster analysis for MD simulations A and C at t=0 and t=500 ns.
